# Risk Factors of Pancreatic Cancer in Patients With Type 2 Diabetes Mellitus: The Hong Kong Diabetes Study

**DOI:** 10.1210/jendso/bvac138

**Published:** 2022-09-05

**Authors:** Raymond Ngai Chiu Chan, Teddy Tai Loy Lee, Oscar Hou In Chou, Jenny So, Cheuk To Chung, Edward Christopher Dee, Kenrick Ng, Pias Tang, Leonardo Roever, Tong Liu, Wing Tak Wong, Gary Tse, Sharen Lee

**Affiliations:** Diabetes Research Unit, Cardiovascular Analytics Group, Hong Kong, China–UK Collaboration, Hong Kong, China; Diabetes Research Unit, Cardiovascular Analytics Group, Hong Kong, China–UK Collaboration, Hong Kong, China; Diabetes Research Unit, Cardiovascular Analytics Group, Hong Kong, China–UK Collaboration, Hong Kong, China; Diabetes Research Unit, Cardiovascular Analytics Group, Hong Kong, China–UK Collaboration, Hong Kong, China; Diabetes Research Unit, Cardiovascular Analytics Group, Hong Kong, China–UK Collaboration, Hong Kong, China; Department of Radiation Oncology, Memorial Sloan Kettering Cancer Center, New York, New York, USA; Department of Medical Oncology, University College London Hospitals NHS Foundation Trust, London, UK; Diabetes Research Unit, Cardiovascular Analytics Group, Hong Kong, China–UK Collaboration, Hong Kong, China; Department of Clinical Research, Federal University of Uberlandia, Uberlandia, Brazil; Tianjin Key Laboratory of Ionic-Molecular Function of Cardiovascular Disease, Department of Cardiology, Tianjin Institute of Cardiology, Second Hospital of Tianjin Medical University, Tianjin 300211, China; School of Life Sciences, The Chinese University of Hong Kong, Hong Kong, China; Tianjin Key Laboratory of Ionic-Molecular Function of Cardiovascular Disease, Department of Cardiology, Tianjin Institute of Cardiology, Second Hospital of Tianjin Medical University, Tianjin 300211, China; Kent and Medway Medical School, University of Kent and Canterbury Christ Church University, Canterbury, CT2 7NT, UK; Diabetes Research Unit, Cardiovascular Analytics Group, Hong Kong, China–UK Collaboration, Hong Kong, China

**Keywords:** diabetes, pancreatic cancer, chronic kidney disease, diabetic microvascular complications

## Abstract

**Context:**

Diabetes mellitus (DM) is associated with the development of pancreatic cancer (PaC), but few large-scale studies have examined its predictive risk factors.

**Objective:**

The present study aims to examine the predictors for PaC in patients with type 2 diabetes mellitus (T2DM) in a territory-wide, retrospective cohort study.

**Methods:**

This was a territory-wide, retrospective cohort study of patients with T2DM mellitus older than 40 years with no prior history of PaC. Baseline demographics, use of antidiabetic medications, comorbidities, and biochemical parameters were extracted. Cox regression was used to calculate hazard ratios (HR) with 95% CI. Subgroup analyses based on chronic kidney disease (CKD) stages were performed.

**Results:**

This study consisted of 273 738 patients (age = 65.4 ± 12.7 years, male = 48.2%, follow-up duration = 3547 ± 1207 days, disease duration = 4.8 ± 2.3 years), of whom 1148 developed PaC. The number of antidiabetic medications prescribed (HR: 1.20; 95% CI, 1.01-1.42; *P* = .040), diabetic microvascular complications (HR: 1.91; 95% CI, 1.30-2.81; *P* < .001), chronic kidney disease (HR: 1.81; 95% CI, 1.25-2.64; *P* = .002), use of acarbose (HR: 2.24; 95% CI, 1.35-3.74; *P* = .002), and use of glucagon-like peptide-1 receptor agonist (HR: 4.00; 95% CI: 1.28-12.53, *P* = .017) were associated with PaC development on multivariable Cox regression adjusting for the duration of DM, mean glycated hemoglobin A_1c_, and history of pancreatic diseases. Stage 3A CKD or below was associated with PaC but not stage 3B or beyond.

**Conclusion:**

Diabetic microvascular complications, especially stage 1, 2, and 3A CKD, were associated with PaCs.

Diabetes mellitus (DM) is a global public health concern currently affecting around 463 million patients worldwide. Current estimates predict a further rise of 51% by 2045 [1]. DM has long been associated with a number of microvascular and macrovascular complications. In recent years, more attention has been drawn to the association between DM and various cancers [2–4]. Several meta-analyses were published establishing the relations between DM and many common malignancies including lung cancer, gastric cancer, colorectal cancer, hepatocellular carcinoma, breast cancer, as well as cancer mortality [5–10].

Pancreatic cancer (PaC), a relatively rare yet highly aggressive malignancy, is associated with a dismal prognosis and has a 5-year-survival rate of less than 5% [11]. It is currently the third leading cause of cancer-related death in the United States [12]. Despite advances in oncological treatments over previous decades, it remains a growing source of cancer death, potentially because of difficulty in diagnosis and delayed treatment. Identifying patients at risk of developing PaC and subsequent workups may hence be important to improve the prognosis.

Meanwhile, PaC has been associated with DM [13–15]. In a meta-analysis of 35 cohort studies in 2011, DM was identified as a risk factor of PaC independent of sex, alcohol consumption, body mass index, and smoking [16]. In another meta-analysis conducted in 2015, Song et al [17] pooled data from 44 studies and further reported that a long DM duration was associated with an elevated risk of PaC of up to 64%. In addition, DM predicts not only incident PaC, but also PaC-related mortality. A large-scale cohort study of 1 089 586 individuals in the United States reported an adjusted risk ratio of up to 2.05 for PaC mortality in DM patients compared to those without DM [18].

However, although DM patients are at higher risks of developing PaC, risk stratification is imperfect as there is limited literature reporting the potential risk factors of PaC in DM patients thus far. Therefore, the present study aims to examine the predictors for PaC in patients with type 2 DM (T2DM) in a territory-wide, retrospective cohort study.

## Materials and Methods

### Study Design and Data Source

This study was approved by the Joint Chinese University of Hong Kong-New Territories East Cluster Clinical Research Ethics Committee. It was a population-based, territory-wide, retrospective cohort study with the data from the Clinical Data Analysis and Reporting System (CDARS), an electronic medical database that captures inpatient and outpatient data from all public hospitals and clinics in Hong Kong, China. CDARS facilitates the retrieval of clinical data from differential operation systems in health care institutes to provide integrated clinical data and represents approximately 80% of the population [19]. With the use of CDARS, local teams have conducted numerous epidemiological studies previously, including those on DM [19–23]. Confidentiality was ensured by anonymization of data from all subjects.

### Participants and Data Collection

Patients with T2DM older than 40 years from January 1, 2009 to December 31, 2009, were identified. DM was defined by the International Classification of Disease, Ninth Revision (ICD-9) coding system and/or exposure to any antihyperglycemic agents. Diabetes disease duration was defined as the date difference between the earliest of 1) earliest record of T2DM-related ICD-9 codes; 2) earliest record of glycated hemoglobin A_1c_ (HbA_1c_) greater than 6.5%; and 3) earliest record of fasting blood glucose (FBG) greater than 7 mmol/L and the baseline date. Baseline data were defined as data obtained closest to January 1, 2009. Individuals with a history of PaC before baseline were excluded. PaC was defined by the ICD-9 coding system. All participants were followed from baseline until the date of PaC, registered death, last follow-up, or August 16, 2020, whichever came first.

Demographic information including date of birth, age, sex, and date of registered death was captured. The following classes of antidiabetic medications were extracted: 1) biguanide; 2) sulfonylurea; 3) insulin; 4) thiazolidinedione; 5) alpha-glucosidase inhibitor; and 6) glucagon-like peptide-1 receptor agonist (GLPA). The use of these agents was defined as any exposure at or before baseline.

At baseline, laboratory data from complete blood count (lymphocyte count, neutrophil count, and hemoglobin level), liver function test (alanine transaminase, alkaline phosphatase [ALP), albumin, and total protein), renal function test (creatinine, urea, and estimated glomerular filtration rate [eGFR]), lipid profile (high-density lipoprotein cholesterol (HDL), low-density lipoprotein cholesterol (LDL-), total cholesterol, triglycerides), and glycemic profile (FBG, HbA_1c_) between January 1, 2008 to December 31, 2008, were obtained. In addition, mean HbA_1c_ and FBG from January 1, 2008 to December 31, 2008, were extracted. Baseline anemia was defined as hemoglobin count less than 13 g/dL among men, and less than 12 g/dL among women.

The following preexisting comorbidities were identified using ICD-9 codes (Supplementary Table 1) [24]: 1) diabetic retinopathy; 2) diabetic neuropathy; 3) peripheral vascular disease; 4) ischemic stroke; 5) sudden cardiac death; 6) atrial fibrillation; 7) heart failure (HF); 8) ischemic heart disease; 9) hypertension; 10) dementia; 11) cancer; 12) depression; 13) acute pancreatitis; 14) chronic pancreatitis; 15) pancreatic cyst. Chronic kidney disease (CKD) was defined by ICD-9 or an eGFR less than 90 mL/min/1.73 m^2^. Stage < 2, 3A, 3B, 4, and 5 CKD were defined as eGFR greater than 60, 59 to 45, 44 to 30, 29 to 15, and less than 15 mL/min/1.73 m^2^, respectively. Hyperlipidemia was defined by ICD-9, triglycerides greater than 1.7 mmol/L or LDL greater than 3.4 mmol/L. Diabetic microvascular complications were defined as CKD, diabetic retinopathy, or diabetic neuropathy. Diabetic macrovascular complications were defined as peripheral vascular disease, ischemic stroke, sudden cardiac death, atrial fibrillation, HF, or ischemic heart disease. Pancreatic disease was defined by ICD-9, acute pancreatitis, chronic pancreatitis, or benign pancreatic cyst.

### Statistical Analysis

Data were analyzed using R Studio software (version 1.1.456). Continuous variables were expressed as mean ± SD while categorical variables were expressed as frequency (percentage). Qualitative and quantitative differences between participants who developed PaC and those who did not were analyzed using chi-square or Fisher exact tests for categorical data and *t* test, Mann-Whitney test, one-way analysis of variance, and Kruskal-Wallis test for continuous data, where appropriate. Unadjusted hazard ratios (HRs) were calculated using a univariable Cox regression model after censoring for death. It is presented with 95% CI. Variables with *P* less than .05 will be included in the multivariable analysis. Adjusted HRs were then calculated using multivariable Cox regression after censoring for death. We constructed 3 multivariable models to adjust for DM duration, DM duration and mean HbA_1c_, as well as DM duration, mean HbA_1c_, and pancreatic diseases respectively. Univariable and multivariable Cox regression was performed to assess the association of different stages of CKD and PaC. All statistical tests were 2-sided and statistical significance was taken as *P* less than .05.

## Results

### Baseline Characteristics

A total of 273 738 participants with DM were identified after excluding individuals with PaC before baseline (age = 65.4 ± 12.7 years, male = 48.2%, follow-up duration = 3547 ± 1207 days, DM duration = 4.8 ± 2.3 years). Their mean FBG and HbA_1c_ were 8.00 ± 1.86 mmol/L and 7.75 ± 2.6%, respectively. A total of 37.0% and 15.3% patients had microvascular and macrovascular complications at baseline; 67.9%, 63.4% and 10.8% patients were on metformin, sulfonylurea, and insulin, respectively. During follow-up, 33.3% of patients died. The demographic information of participants who developed and did not develop PaC during follow-up is summarized in Table 1.

**Table 1.** Baseline characteristics of individuals who developed and did not develop pancreatic cancer

	No pancreatic cancer (n = 272 590)	Pancreatic cancer (n = 1148)	*P*
**Basic demographics**			
**Male**	**131 397 (48.2)**	**562 (49.0)**	.**632**
**Age, y**	**65.4 (12.7)**	**69.1 (10.2)**	**<** .**001**
**Follow-up duration, d**	**3552.0 (1204.3)**	**2246 (1253)**	**<** .**001**
**DM duration, y**	**4.8 (2.3)**	**4.8 (2.2)**	.**872**
**HbA_1c_, %**	**7.4 (1.5)**	**7.4 (1.3)**	**<** .**001**
**Mean HbA_1c_**	**7.7 (1.2)**	**7.75 (1.16)**	.**163**
**Fasting blood glucose**	**7.8 (2.6)**	**7.59 (2.39)**	.**123**
**Mean fasting blood glucose**	**8.0 (1.9)**	**7.83 (1.68)**	.**005**
**No. of DM medications**	**1.4 (0.8)**	**1.5 (0.8)**	.**036**
**Death**	**89 993 (33.0)**	**1063 (93.6)**	**<** .**001**
**Microvascular complications**	**100 770 (37.0)**	**462 (40.2)**	**<** .**001**
**CKDs**	**100 366 (36.8)**	**460 (40.1)**	**<** .**001**
**Diabetic neuropathy**	**1159 (0.4)**	**3 (0.3)**	.**532**
**Diabetic retinopathy**	**3531 (1.3)**	**12 (1.0)**	.**537**
**Stage of CKD**			**<** .**001**
**< 2**	**56 808 (20.8)**	**283 (24.7)**	
**3A**	**21 249 (7.8)**	**109 (9.5)**	
**3B**	**13 131 (4.8)**	**48 (4.2)**	
**4**	**6025 (2.2)**	**15 (1.3)**	
**5**	**2885 (1.1)**	**4 (0.3)**	
**Macrovascular complication**	**41 796 (15.3)**	**159 (13.9)**	.**177**
**Peripheral vascular diseases**	**346 (0.1)**	**0 (0.0)**	.**429**
**Ischemic stroke**	**8950 (3.3)**	**27 (2.4)**	.**092**
**Sudden cardiac death**	**6391 (2.3)**	**26 (2.3)**	.**936**
**Atrial fibrillation**	**7744 (2.8)**	**23 (2.0)**	.**106**
**Heart failure**	**11 146 (4.1)**	**30 (2.6)**	.**014**
**Coronary heart disease**	**26 301 (9.6)**	**106 (9.2)**	.**671**
**Pancreatic diseases**	**510 (0.2)**	**18 (1.6)**	**<** .**001**
**Acute pancreatitis**	**275 (0.1)**	**3 (0.3)**	.**215**
**Chronic pancreatitis**	**110 (0.0)**	**5 (0.4)**	**<** .**001**
**Pancreatic cyst**	**119 (0.0)**	**2 (0.2)**	.**163**
**Other comorbidities**			
**Hypertension**	**63 941 (23.5)**	**259 (22.6)**	.**497**
**Hyperlipidemia**	**78 593 (28.8)**	**312 (27.2)**	.**143**
**Dementia**	**2833 (1.0)**	**8 (0.7)**	.**319**
**Other cancers**	**11 994 (4.4)**	**58 (5.1)**	.**316**
**Anemia**	**19 317 (7.1)**	**74 (6.4)**	.**432**
**Depression**	**4350 (1.6)**	**18 (1.6)**	**≥ .999**
**Liver function test**			
**ALP**	**79.8 (36.3)**	**83.8 (66.2)**	.**173**
**ALT**	**26.1 (24.4)**	**26.6 (27.8)**	.**708**
**Total protein**	**74.2 (7.0)**	**74.2 (6.0)**	.**873**
**Albumin**	**38.7 (5.4)**	**39.1 (4.9)**	.**064**
**Complete blood count**			
**Hemoglobin**	**12.4 (2.0)**	**12.6 (1.9)**	.**249**
**Lymphocytes**	**1.8 (1.1)**	**1.8 (0.8)**	.**436**
**Neutrophils**	**5.5 (2.8)**	**5.2 (2.5)**	.**221**
**Lipid profile**			
**HDL**	**1.2 (0.3)**	**1.2 (0.3)**	.**437**
**LDL**	**2.9 (0.9)**	**2.9 (0.8)**	.**443**
**Total cholesterol**	**4.8 (1.0)**	**4.7 (1.0)**	.**016**
**Triglycerides**	**1.7 (1.4)**	**1.6 (1.0)**	.**020**
**Renal function test**			
**Creatinine**	**103.6 (93.7)**	**92.2 (48.0)**	**<** .**001**
**Urea**	**6.9 (4.1)**	**6.4 (2.9)**	**<** .**001**
**eGFR**	**70.1 (24.8)**	**70.1 (20.8)**	.**978**
**DM medications**			
**Biguanide**	**184 999 (67.9)**	**814 (70.9)**	.**030**
**Sulfonylurea**	**172 693 (63.4)**	**742 (64.6)**	.**385**
**Insulin**	**29 540 (10.8)**	**120 (10.5)**	.**711**
**Pioglitazone**	**3680 (1.4)**	**18 (1.6)**	.**610**
**Alpha-glucosidase inhibitor**	**3265 (1.2)**	**23 (2.0)**	.**018**
**GLPA**	**336 (0.1)**	**4 (0.3)**	.**082**

Abbreviations: ALP, alkaline phosphatase; ALT, alanine transaminase; CKD, chronic kidney disease; DM, diabetes mellitus; eGFR, estimated glomerular filtration rate; HbA_1c_, glycated hemoglobin A_1c_; HDL, high-density lipoprotein cholesterol; LDL, low-density lipoprotein cholesterol; GLPA, glucagon-like peptide-1 receptor agonist.

Of the 273 738 patients, 1148 (0.4%) developed PaC over a mean follow-up of 2246 ± 1253 days. Compared to patients who did not develop PaC, they were older, had higher frequencies of microvascular complications, CKD, HF, pancreatic diseases, and acute pancreatitis at baseline. They were on a higher number of antidiabetic medications, especially metformin and acarbose. Of the patients who developed incident PaC, 93.6% patients died during follow-up within the study period, reflecting the dismal prognosis of this cancer.

### Pancreatic Diseases in Diabetic Mellitus Patients

Univariable Cox regression was used to identify the potential risk factors of PaC development. Pancreatic diseases were the strongest predictor of PaC with an unadjusted HR of 32.68 (95% CI, 18.05-59.18; *P* < .001). Of the 3 pancreatic diseases analyzed, chronic pancreatitis and pancreatic cyst were associated (Table 2). The association between pancreatic diseases and chronic pancreatitis remains statistically significant after adjustment by DM duration and mean HbA_1c_ (Table 3).

**Table 2.** Unadjusted hazard ratio for developing pancreatic cancer in diabetes mellitus patients

	Unadjusted HR	95% CI	*P*
**DM duration**	**1**.**01**	**0.97-1.04**	.**779**
**HbA_1c_**	**0**.**96**	**0.90-1.03**	.**244**
**Fasting blood glucose**	**0**.**97**	**0.94-1.01**	.**16**
**Mean HbA_1c_**	**1**.**06**	**0.99-1.14**	.**115**
**Mean fasting blood glucose**	**0**.**933**	**0.89-0.98**	.**009**
**DM drug number**	**1**.**09**	**1.01-1.17**	.**022**
**Microvascular complication**	**1**.**72**	**1.37-2.15**	**<** .**001**
**Chronic kidney disease**	**1**.**70**	**1.36-2.12**	**<** .**001**
**Diabetic neuropathy**	**0**.**76**	**0.24-2.35**	.**628**
**Diabetic retinopathy**	**0**.**96**	**0.54-1.69**	.**874**
**Macrovascular complications**	**1**.**06**	**0.89-1.25**	.**526**
**Ischemic stroke**	**0**.**86**	**0.59-1.26**	.**431**
**Sudden cardiac death**	**1**.**16**	**0.79-1.71**	.**451**
**Atrial fibrillation**	**0**.**90**	**0.60-1.37**	.**632**
**Heart failure**	**0**.**91**	**0.63-1.30**	.**590**
**Coronary heart disease**	**1**.**09**	**0.89-1.33**	.**411**
**Pancreatic diseases**	**32**.**68**	**18.05-59.18**	**< .001**
**Acute pancreatitis**	**2**.**82**	**0.91-8.74**	.**074**
**Chronic pancreatitis**	**12**.**07**	**5.02-29.06**	**< .001**
**Pancreatic cyst**	**4**.**23**	**1.06-16.93**	.**042**
**Hypertension**	**1**.**09**	**0.95-1.25**	.**239**
**Hyperlipidemia**	**0**.**85**	**0.72-1.02**	.**076**
**Dementia**	**1**.**06**	**0.53-2.12**	.**879**
**Other cancers**	**1**.**42**	**1.09-1.85**	.**009**
**Anemia**	**1**.**28**	**1.01-1.61**	.**041**
**Depression**	**1**.**02**	**0.64-1.63**	.**93**
**ALP**	**1**.**00**	**1.00-1.00**	**< .001**
**ALT**	**1**.**00**	**1.00-1.00**	.**997**
**Total protein**	**0**.**99**	**0.97-1.01**	.**182**
**Albumin**	**0**.**99**	**0.97-1.02**	.**643**
**Hemoglobin**	**0**.**97**	**0.89-1.05**	.**414**
**Lymphocytes**	**0**.**98**	**0.79-1.20**	.**819**
**Neutrophils**	**0**.**99**	**0.93-1.05**	.**703**
**HDL**	**1**.**07**	**0.86-1.34**	.**600**
**LDL**	**0**.**95**	**0.85-1.06**	.**339**
**Total cholesterol**	**0**.**90**	**0.84-0.97**	.**004**
**Triglycerides**	**0**.**93**	**0.87-1.00**	.**041**
**Creatinine**	**1**.**00**	**1.00-1.00**	.**236**
**Urea**	**0**.**99**	**0.96-1.01**	.**354**
**eGFR**	**1**.**00**	**0.99-1.00**	.**008**
**Stage of CKD**	**1**.**07**	**1.00-1.16**	.**053**
**Metformin**	**1**.**08**	**0.95-1.23**	.**244**
**Sulfonylurea**	**1**.**08**	**0.96-1.22**	.**192**
**Insulin**	**1**.**09**	**0.90-1.31**	.**390**
**Pioglitazone**	**1**.**13**	**0.71-1.18**	.**615**
**Acarbose**	**1**.**76**	**1.16-2.65**	.**008**
**GLPA**	**2**.**67**	**1.00-7.12**	.**050**

Abbreviations: ALP, alkaline phosphatase; ALT, alanine transaminase; CKD, chronic kidney disease; DM, diabetes mellitus; eGFR, estimated glomerular filtration rate; HbA_1c_, glycated hemoglobin A_1c_; HDL, high-density lipoprotein cholesterol; HR, hazard ratio; LDL, low-density lipoprotein cholesterol; GLPA, glucagon-like peptide-1 receptor agonist.

**Table 3.** Adjusted hazard ratio for developing pancreatic cancer in diabetes mellitus patients

	Model 1	Model 2	Model 3
**Mean fasting blood glucose**	**0.93 (0.87-1.00)** ***P* = .040**	**0.88 (0.81-0.96)** ***P* = .004**	**0.88 (0.81-0.96)** ***P* = .004**
**No. of DM medications**	**1.06 (0.96-1.17)** ***P* = .256**	**1.20 (1.01-1.42)** ***P* = .041**	**1.20 (1.01-1.42)** ***P* = .040**
**Microvascular complications**	**1.78 (1.32-2.40)** ***P* < .001**	**1.91 (1.30-2.81)** ***P* < .001**	**1.91 (1.30-2.81)** ***P* < .001**
**Chronic kidney disease**	**1.73 (1.29-2.31)** ***P* < .001**	**1.81 (1.25-2.64)** ***P* = .002**	**1.81 (1.25-2.64)** ***P* = .002**
**Pancreatic diseases**	**5.13 (2.29-11.45)** ***P* < .001**	**2.05 (0.50-8.25)** ***P* = .313**	**N/A**
**Chronic pancreatitis**	**12.44 (4.00-38.67)** ***P* < .001**	**11.45 (2.84-46.14)** ***P* < .001**	**N/A**
**Pancreatic cyst**	**6.71 (1.67-26.88)** ***P* = .003**	**0.00 (0.00-inf)** ***P* = .990**	**N/A**
**Other cancers**	**1.43 (1.02-2.01)** ***P* = .039**	**1.58 (1.00-2.48)** ***P* = .050**	**1.57 (1.00-2.48)** ***P* = .051**
**Anemia**	**1.21 (0.89-1.65)** ***P* = .224**	**1.01 (0.70-1.47)** ***P* = .952**	**1.01 (0.70-1.46)** ***P* = .976**
**ALP**	**1.00 (1.00-1.01)** ***P* < .001**	**1.00 (1.00-1.01)** ***P* = .036**	**1.00 (1.00-1.01)** ***P* = .040**
**Total cholesterol**	**0.93 (0.84-1.02)** ***P* = .111**	**0.97 (0.86-1.09)** ***P* = .584**	**0.97 (0.86-1.08)** ***P* = 0.583**
**Triglycerides**	**0.99 (0.92-1.07)** ***P* = .740**	**1.01 (0.93-1.09)** ***P* = .839**	**1.01 (0.93-1.09)** ***P* = .873**
**Alpha-glucosidase inhibitor**	**2.04 (1.24-3.35)** ***P* = .005**	**2.24 (1.34-3.73)** ***P* = .002**	**2.24 (1.35-3.74)** ***P* = .002**
**GLPA**	**4.54 (1.70-12.13)** ***P* = .003**	**3.99 (1.27-12.47)** ***P* = .018**	**4.00 (1.28-12.53)** ***P* = .017**

Model 1: Adjusted for DM duration; model 2: adjusted for DM duration and mean HbA_1c_; model 3 adjusted for DM duration, mean HbA_1c_, and pancreatic diseases.

Abbreviations: ALP, alkaline phosphatase; DM, diabetes mellitus; GLPA, glucagon-like peptide-1 receptor agonist; HbA_1c_, glycated hemoglobin A_1c_; N/A, not available.

### Diabetic Complications and Comorbidities

Diabetic microvascular complications were associated with a higher risk of developing PaC during follow-up (HR: 1.72; 95% CI, 1.37-2.15; *P* < .001) (see Table 2). Among the 3 microvascular complications, only CKD was associated with PaC (HR: 1.70; 95% CI, 1.36-2.12; *P* < .001). The association for both diabetic microvascular complications and CKD remained statistically significant after adjustment for DM duration, mean HbA_1c_, and pancreatic diseases (see Table 3). Given the association of CKD and PaC on multivariable regression, we further analyzed the association of the stage of CKD and eGFR with the development of PaC, but neither showed statistically significant associations (see Table 2). Patients were stratified according to CKD stage. Patients with less than stage 2 CKD were at greater risk of PaC after adjusting for DM duration, mean HbA_1c_, and pancreatic diseases (HR: 1.91; 95% CI, 1.28-2.83; *P* = .001). Stage 3A CKD showed an even stronger association with a, HR of 2.16 (95% CI, 1.38-3.39; *P* < .001) (Table 4). No statistically significant association was found for any macrovascular complications (HR: 1.06; 95% CI, 0.89-1.25; *P* = .526) (see Table 2). A history of malignancy remained a statistically significant predictor for PaC (HR: 1.57; 95% CI, 1.00-2.48; *P* = .050) after adjusting for DM duration and mean HbA_1c_ (see Table 3).

**Table 4.** Association of stages of chronic kidney disease and pancreatic cancer in diabetes mellitus patients

	Unadjusted HR	Model 1	Model 2	Model 3
**< Stage 2**	1.73 (1.37-2.18) *P* < .001	1.80 (1.33-2.45) *P* < .001	1.90 (1.28-2.82) *P* = .001	1.91 (1.28-2.83) *P* = .001
**Stage 3A**	1.91 (1.45-2.52) *P* < .001	1.98 (1.39-2.84) *P* < .001	2.16 (1.38-3.38) *P* < .001	2.16 (1.38-3.39) *P* < .001
**Stage 3B**	1.50 (1.06-2.13) *P* = .021	1.27 (0.78-2.04) *P* = .302	1.36 (0.77-2.41) *P* = .287	1.36 (0.77-2.41) *P* = .286
**Stage 4**	1.22 (0.71-2.12) *P* = .469	1.03 (0.49-2.18) *P* = .931	1.35 (0.62-2.95) *P* = .449	1.35 (0.62-2.93) *P* = .456
**Stage 5**	0.92 (0.34-2.51) *P* = .873	0.76 (0.18-3.14) *P* = .706	0.96 (0.23-4.02) *P* = .950	0.96 (0.23-4.04) *P* = .955

Model 1: Adjusted for DM duration; model 2: adjusted for DM duration and mean HbA_1c_; model 3 adjusted for DM duration, mean HbA_1c_, and pancreatic diseases.

Abbreviations: DM, diabetes mellitus; HbA_1c_, glycated hemoglobin A_1c_; HR, hazard ratio.

### Biochemical Parameters

Among all the parameters, only eGFR, ALP, total cholesterol, and triglycerides were associated with PaC in univariable analyses (see Table 2). The association of eGFR, total cholesterol, and triglycerides with PaC became insignificant after adjusting for DM duration (see Table 3). Only ALP produced a statistically significant association with PaC after adjustment for DM duration, mean HbA_1c_, and pancreatic diseases, but the association was very weak (HR: 1.00; 95% CI, 1.00-1.01; *P* = .040).

### Diabetic Control and Medications

Among the parameters of baseline diabetic control, only the number of DM medications produced a statistically significant association (HR: 1.09; 95% CI, 1.01-1.17; *P* = .022) (see Table 2). The association remained statistically significant after adjusting for DM duration, mean HbA_1c_, and pancreatic diseases (see Table 3). GLPA (HR: 2.67; 95% CI, 1.00-7.12; *P* = .050) and alpha-glucosidase inhibitor (HR: 1.76; 95% CI, 1.16-2.65; *P* = .008) were associated with PaC both in univariable and multivariable analyses adjusting for DM duration, mean HbA_1c_, and pancreatic diseases.

## Discussion

This was a territory-wide, retrospective cohort study that aimed to identify the risk factors of developing PaC in DM patients, and is thus far one of the largest cohort studies for PaC specific to DM individuals that included demographic data, DM control, use of medications, and comorbidities at baseline for analysis. In our study, the following important findings were noted: 1) DM duration and diabetic control were not associated with risk of PaC; 2) no baseline biochemical parameters can predict PaC; 3) microvascular complications, in particular CKD, were strong predictors of PaC; and 4) the use of acarbose and GLP-based medications were associated statistically.

### Diabetic Duration and Control

DM has been recognized as a risk factor of PaC by multiple studies and more recently several meta-analyses [13, 14, 16, 17, 25, 26]. Although there have been studies reporting the association between long DM duration and PaC, in our study we demonstrated that DM duration was not associated. While the meta-analysis in 2015 concluded that “long-term” DM was associated with an increased risk of PaC, findings from the present study echoed their findings in that a positive relation between DM duration and risk of PaC was not observed [17]. In their study, DM duration of 2 or more years was associated with a 64% increase in PaC risk, while the pooled risk ratio was only 1.50 for DM duration of 10 or more years. Similar data were also presented by Ben et al [16], who reported that patients with DM duration greater than 1 year had a relative risk greater than patients with DM duration greater than 5 years, showing that a longer DM duration did not result in a higher risk of PaC.

Meanwhile, for DM control, our data were similar to another large-scale population-based study consisting of 1 574 768 individuals in Sweden that showed that baseline HbA_1c_ at DM diagnosis was not associated with PaC [27]. We further evaluated the effect of mean HbA_1c_ to account for variability in only one measurement and the result was similar. Although a sudden increase in HbA_1c_, that is, new-onset DM, may be a symptom of PaC indicating that in case-control studies a higher HbA_1c_ was associated with PaC [28], this phenomenon was not observed by both population-based studies when PaC was relatively uncommon. Contrary to our findings, a meta-analysis showed that FBG was associated with PaC. However, their study included only 576 cases from 5 studies, and the heterogeneity was high between studies [29]. It is likely that glycemic control in patients with established DM was not associated with PaC.

### Diabetic Complications and Pancreatic Cancer

In a nationwide, population-based study in Korea, predialytic CKD including hypertensive nephropathy, diabetic nephropathy, and unspecified kidney failure was shown to be associated with PaC compared to the general population [30]. However, that study did not adjust for DM or other established risk factors of PaC. Whether CKD is an independent risk factor in the general or the DM population remains unclear. To the best of our knowledge, our present study is the first population-based cohort study to identify CKD as a risk factor of PaC in DM patients. This echoed the findings from a small-scale case-control study that included 433 PaC patients from a multiethnic cohort [31]. In both studies, CKD was significantly associated with PaC but not other microvascular complications including diabetic retinopathy or neuropathy. As described in previous studies, DM and hyperglycemia contributed to the risk of PaC by attenuating antioxidant enzyme activity, and upregulating cellular levels of free radicals [32]. The role of advanced glycation products has also been implicated, given the fact that their receptors bind several important ligands in inflammation and carcinogenesis [33].

The present study showed that in addition to DM and hyperglycemia, CKD further elevated the risk of PaC. This may be a result of multiple factors including vitamin D deficiency, uremia, the proinflammatory state, and oxidative stress in CKD as proposed by previous studies [34–38]. Shortened leukocyte telomere length, which has been proposed to be a marker of oxidative stress and cellular aging, was also shown to be associated with both CKD and PaC [38, 39], highlighting their potential mechanistic relations. In addition to these shared pathways, CKD may also potentiate the effects of hyperglycemia on PaC development as exogenous or endogenous uremic toxins, angiotensin II, iron overload, and lipoperoxides create substantial amount of reactive oxygen species, which in turn upregulates advanced glycation products receptors expression via inhibition of glyceraldehyde-3-phosphate dehydrogenase and accumulation of upstream glycolytic intermediates [40, 41]. Such an association, together with the difficulty in diagnosing PaC in early stages, should alert physicians to the possibility of PaC whenever gastrointestinal symptoms arise in DM patients with CKD.

After identifying the association between CKD and PaC, we further analyzed the association of PaC with stage of CKD and eGFR respectively, but no statistically significant results were produced. Unadjusted and adjusted HRs were then calculated for each stage of CKD as compared to patients without CKD. Only stages before 3A were associated with PaC after adjustment but not stage 3B or above. The relatively fewer individuals having advanced stages of CKD and the shorter life expectancy may account for the loss of association beyond stage 3B and potentially explained the lack of association between eGFR and stages of CKD with PaC.

### Antidiabetic Medication and Pancreatic Cancer

In the present study, we demonstrated that acarbose and GLPA were associated with PaC statistically. For GLPA, there have been historical concerns regarding the risk of PaC in earlier observational studies [42]. Subsequently, several randomized controlled trials and meta-analyses were conducted, which confirmed the safety of GLPA [43]. The observed association between GLPA and PaC in previous studies was thought to be a result of confounding factors, which also potentially existed in the present study. Meanwhile, data on the risk of PaC in acarbose users were more scarce. Although a nationwide study in South Korea showed a small increase in the risk of PaC among alpha-glucosidase inhibitor users after adjustment [44], a meta-analysis of 4 studies reported no statistically significant associations [45]. Owing to the weight reduction effect, we postulate that GLPA and acarbose were more frequently prescribed in patients with higher body mass index, a traditional risk factor that we were not able to adjust for because of the retrospective nature of our study [46, 47]. Hence, the statistical association shown in the present study should be interpreted cautiously and further investigation is warranted.

### Strengths and Limitations

With the use of data from CDARS, which represented approximately 80% of the local population, the present study had an adequate sample size and follow-up duration to detect relatively rare outcomes, such as PaC. It was also sufficiently powered to identify the association between PaC and different characteristics of the participants, including the different stages of CKD, which may not be possible for smaller cohorts. Nonetheless, there are still several limitations in our study. First, although CDARS has been recognized as a reliable source of integrated clinical data and has been used to produce numerous quality studies, there was unavoidably a risk of undercoding and coding errors. To partially compensate for the problems in coding, we captured several biochemical parameters, for example, FBG and HbA_1c_, baseline creatinine and eGFR, triglycerides, and LDL, as well as hemoglobin, to define DM and its comorbidities like CKD, hyperlipidemia, and anemia. Second, data on DM duration and PaC were based on the date of the initial diagnosis in CDARS, which may or may not correspond to the actual date of diagnosis, especially if patients were diagnosed before January 1, 2009. Third, the relatively large number of individuals potentially resulted in high statistical significance but low HR in certain predictive parameters. The clinical significance may hence be limited. Fourthly, c-peptide was not measured in all individuals. Participants with DM secondary to pancreatic diseases may be misclassified as T2DM. However, the overall number of patients with pancreatic diseases was very small and was unlikely to have major impacts on subsequent analyses. Finally, data on certain traditional risk factors of PaC, for example, family history, smoking, alcohol consumption, and obesity [48], were not available because of limitations of our database.

## Conclusion

Early-stage CKD, a microvascular complication, significantly predicted PaC after adjusting for DM duration, mean HbA_1c_, and pancreatic diseases, but not macrovascular complications. Further studies are required to establish the relations of these potential risk factors, and risk stratification of DM patients based on a combination of patient demographic, comorbidities, and use of medications is crucial.

## Financial Support

The authors received no financial support for the research, authorship, and/or publication of this article.

## Author Contributions

R.N.C.C.: study conception, data processing, data interpretation, statistical analysis, manuscript drafting, and critical revision of the manuscript; T.T.L.L. and O.H.I.C.: data acquisition, data processing, data interpretation, manuscript drafting, and critical revision of the manuscript; J.S., C.T.C., E.C.D., K.N., P.T., L.R., T.L., and W.T.W.: study planning, data interpretation, and critical revision of the manuscript; G.T. and S.L.: study conception, data processing, data interpretation, manuscript drafting, critical revision of the manuscript, and study supervision.

## Disclosures

The authors have nothing to disclose.

## Data Availability

Restrictions apply to the availability of some or all data generated or analyzed during this study to preserve patient confidentiality or because they were used under license. The corresponding author will on request detail the restrictions and any conditions under which access to some data may be provided.

## Abbreviations

ALP: alkaline phosphatase
CDARS: Clinical Data Analysis and Reporting System
CKD: chronic kidney disease
DM: diabetes mellitus
eGFR: estimated glomerular filtration rate
FBG: fasting blood glucose
GLPA: glucagon-like peptide-1 receptor agonist
HbA_1c_: glycated hemoglobin A_1c_
HDL: high-density lipoprotein cholesterol
HF: heart failure
HR: hazard ratio
ICD-9: International Classification of Diseases Ninth Revision
LDL: low-density lipoprotein cholesterol
PaC: pancreatic cancer
T2DM: type 2 diabetes mellitus
